# Practical School Nutrition Program May Reduce Food Neophobia

**DOI:** 10.3390/nu13103541

**Published:** 2021-10-09

**Authors:** Corinne A. Labyak, Leslie G. Kaplan, Tammie M. Johnson, Meghan Moholland

**Affiliations:** 1 Department of Nutrition and Dietetics, Brooks College of Health, University of North Florida, Jacksonville, FL 32224, USA; c.labyak@unf.edu (C.A.L.); mmoholland94@gmail.com (M.M.); 2 Division of Academic and Student Affairs, Hicks Honors College, University of North Florida, Jacksonville, FL 32224, USA; leslie.kaplan@unf.edu; 3 Institute of Public Health, College of Pharmacy and Pharmaceutical Sciences, Florida A&M University, Tallahassee, FL 32307, USA

**Keywords:** food neophobia, school-based intervention, willingness to taste

## Abstract

The study’s purpose was to evaluate an intervention to reduce fruit and vegetable food neophobia and influence attitudes and behaviors among children using a four-month, non-experimental, before-and-after intervention. Participants were children aged 5–11 years in an intervention school (IS) and a control school (CS). Children were offered fruit or vegetable samples weekly utilizing school-specific psychosocial and educational practices to encourage participation. The outcomes of interest included attitudes measured using a written survey-based food neophobia scale (FNS), behavioral observations, and an oral survey. The post-intervention IS FNS score was significantly lower compared to pre-intervention (*p* = 0.04). Repeated-measures ANOVA revealed a statistically significant overall effect of time (*p* = 0.006). School type-time interaction was not significant (*p* = 0.57). Pre-intervention observational data showed the proportions finishing and taking another fruit and vegetable sample were higher in the CS (*p* < 0.001 for both). Post-intervention, the proportions taking the vegetable (*p* = 0.007) and the fruit (*p* < 0.001) were higher in the IS. The percentage tasting the vegetable was higher in the CS (*p* = 0.009). Offering samples of produce in school lunchrooms may reduce food neophobia. This intervention is an inexpensive program that volunteers can quickly implement.

## 1. Introduction

Several studies suggest that among children, food neophobia, the fear of trying new foods, has deleterious effects on fruit and vegetable consumption and other negative effects on diet [1,2,3,4]. American children consume less than the recommended amount (which varies with age and gender) of vegetables daily (1–4 cups), and average fruit intake (1–2.5 cups) drops significantly below recommendations as children age past nine years [5]. As a result, children are at an increased risk for inadequate micronutrient intake [6,7] and chronic disease [8,9]. Low fruit and vegetable intake is related in all age groups to almost 2.7 million deaths annually worldwide [8]. Dietary patterns are initiated in childhood [10], and thus encouraging a higher fruit and vegetable intake in children is a public health priority. Children with obesity are at greater risk of suffering from depression, anxiety, and bullying [11]. Furthermore, in adulthood, a diet low in fruit and vegetable intake is associated with obesity [12]. Therefore, reducing food neophobia among children, particularly for new fruits and vegetables, could help increase intake [1,3] to encourage a lifelong healthier diet [13] and promote positive health outcomes [5].

School-based interventions have been recommended to increase children’s fruit and vegetable consumption. The USDA National School Lunch program mandated that students with reimbursable lunches must take a fruit and vegetable in hopes of increasing fruit and vegetable intake. Initially the results indicated there was increased waste of these selections by students [14,15]. However, a more recent analysis of the program showed that students selected more fruits and for the students that selected a vegetable, consumption of that vegetable increased [16]. The impact of individual choice remains prevalent despite this intervention, suggesting that food-neophobic children will not choose an unfamiliar option [17] or will not eat it if given, even if the child knows it is healthy. Therefore, efforts to increase opportunities to make healthy choices need to go hand in hand with educational efforts to reduce food neophobia in schools.

There is an abundance of research about locating an educational program in an elementary school to try to increase fruit and vegetable consumption or openness. The World Health Organization catalogued 19 countries that had national programs to encourage people, including children, to eat 3–10 servings of fruit and vegetables per day [18]. The report concluded that factors that determined success included social support and active participation. In addition, longer and more intensive interventions, and those that were school-based, were more successful in the US. Other recommendations for school-based interventions include conducting programs for at least 12 months, exposing the entire school, and utilizing peer encouragement [19], all of which would directly or indirectly address food neophobia. Other reviews of literature that looked at nutrition education interventions including fruit and vegetable intake suggested that the main factors affecting consumption were availability, preparation, and taste preferences [20,21]. One meta-analysis suggests that school-based interventions have a moderate effect on children’s consumption [22], though another qualifies that and suggests that the effect is stronger for fruit than for vegetables [23]. Two other meta-analyses suggest that the particular strategies matter, one focused on experiential learning strategies [24] and the other focused on teacher-delivered programs [25]. The most current research on children’s food neophobia and increasing intake of fruits and vegetables in school-based programs investigates recipe tastings with characters [26], mindfulness activities [27], culinary skills, sensory and nutrition education, food prep, gardening, and going beyond the school setting including the family [28], a game-based program [29], seasonal offerings [30], and using intervention mapping protocol when designing initiatives [31]. Overall, the evidence of previous studies suggests that it is worth exploring nutrition interventions embedded in schools.

The concept of food neophobia is related to the concept of the “omnivore’s dilemma” a psychological theory developed in the 1970s, which posits that omnivores, including humans, require a balance between a willingness to try new foods so that all available resources are used, and an inherent conservatism that protects against ingesting poisons [32]. Researchers in nutrition explored neophobia while documenting children’s preferences and patterns [33,34]. Determining effective interventions to overcome neophobia and increase children’s willingness to try different fruits and vegetables has the potential to improve children’s diet and reduce the impact of chronic disease [5,8,9]. Locating the intervention in a school reaches a large number of children and offers a structured setting in which to implement an intervention, a context that has been examined and found in several meta-analyses to have a moderate impact on fruit and vegetable intake [22,23]. Therefore, the purpose of the current study is to evaluate a weekly fruit and vegetable sampling program that uses a range of psychosocial and educational strategies in an elementary school to reduce neophobia among children. In addition, this study examines differences between attitudes and behavior regarding neophobia.

## 2. Materials and Methods

This study was a 4-month, non-experimental, before-and-after intervention that was based on the results of a 1-year pilot study with a similar design. The unpublished pilot study consisted of tastings in the fall and spring of 2016–17 using the Food Neophobia Scale (FNS) [35] that was adapted for primary school aged children [36]. Although no statistically significant findings were noted, there were relevant changes that supported repeating the study using a larger sample size and the addition of a survey focused more specifically on children’s fruit and vegetable intake. It was approved prior to data collection through the Institutional Review Boards (IRB) at both the University of North Florida and the school district in which the study was conducted. Two elementary schools volunteered to host the study: an intervention and a control school. The two schools were identified using community key informants for elementary schools in the region that were diverse in terms of race, ethnicity and socioeconomic parameters. In each school, classroom teachers were asked to volunteer to participate in the study. Subsequently, students in the first two volunteer classes from each grade (1–5) were invited to participate. Demographic characteristics including gender, age, and race for both schools are included in Table 1. Parents were invited to attend an open meeting to explain the study and obtain parental consent. Teachers also sent home parental consent forms. Student assent was collected in school by the researchers prior to obtaining data. 

The study had two components: (1) administration of the written FVNI survey from which the FNS was calculated, and (2) the fruit and vegetable tasting program. The fruit and vegetable tasting program consisted of offering samples of fruits and vegetables to students in a manner described later in this article. Oral survey responses and observed food-consumption behaviors were documented. At both the intervention and the control schools in study weeks 1 (January) and 9 (April), the Fruit and Vegetable Neophobia Instrument (FVNI) [37], a written survey validated in elementary school aged children, was administered to the selected sample classes in the classroom with the teacher and the researchers present. Appendix A, Table A1 contains the list of questions and response options for the written survey. The intervention was offered over a 4-month period with 9 “study weeks” due to school holidays that interrupted the weekly schedule. The participants who completed the FVNI survey in weeks 1 and 9 varied due to students being out of the classroom at the time of survey administration. This analysis included data from students who: (1) were present during the survey administration for weeks 1 and 9, and (2) had valid responses for all survey questions. These inclusion criteria were selected so that a neophobia scale could be computed and a paired analysis of the neophobia scale could be performed. The observed power was 0.56.

In both schools in weeks 1 and 9, fruit and vegetable neophobia attitudes were assessed through an oral survey, asking students to raise hands if the answer was yes to the following questions: Who has tried this fruit/vegetable before? How many liked it? Disliked it? Unsure? In addition to an oral survey measuring attitudes, researchers conducted behavioral observation, recording the number of students who would take, try, and finish the offered fruit or vegetable and accept a second helping. The oral survey method was chosen for the sake of efficiency because the tastings and oral survey were administered on a weekly basis in the lunchroom where a simple and fast method was necessary. Although there is the possibility of bias as students see the other students raise hands, this was a deliberate strategy of the intervention so that peer examples would encourage more hesitant students to try the weekly sample. Three to 4 research assistants and 1 to 2 principal investigators (PIs) were present at each taste test. Aggregate answers were tallied by research assistants for each classroom and grade level. An a priori power analysis was conducted using an effect size of 0.25. To achieve 0.80 power, it was estimated that a sample size of 70 would be needed for each group. The actual sample sizes exceeded 70 and are provided in Appendix A, Table A2.

Produce was donated by Chartwells, the schools’ food service provider, and each week’s selection was determined by Chartwells, according to availability and cost as well as providing a mix of fruits and vegetables. In both schools, students were given comparable fruits and vegetables in weeks 1 and 9: pears and arugula (week 1) and green apples and spinach (week 9) to be able to measure pre- and post-test experience of both types of produce (comparing fruit to fruit and vegetable to vegetable). Two different leafy greens and 2 different pome fruits were chosen in order that children were offered varieties in the same family to remain comparable but different enough to display neophobia. In the intervention school in weeks 2–8 students were offered 1 sample, either a fruit or a vegetable, and an oral survey and observational data were collected from each research class group. The weekly tasting samples were washed and cut into bite-sized pieces immediately before serving from a large bowl with tongs by the research assistants. In the control school, there was no intervention in weeks 2–8, just the comparable fruits and vegetables in weeks 1 and 9. 

In the middle 7 weeks of the program in the intervention school, the PI’s and research assistants repeated the oral survey and observation and offered all children in the school lunchroom samples of a fruit (cantaloupe, plums, and red apples) or vegetable (green cabbage, orange bell peppers, squash, and broccolini). The samples were offered in a natural context (the lunchroom) to all children in order to not interfere with the social dynamics by singling out the research classes. Research assistants prepared the samples, conducted the oral surveys, and observed and counted the choices made by the children in the research classes to get ongoing behavioral data. 

The psychosocial and educational strategies of the intervention were deployed weeks 2–8 at the intervention school only. Creating a habit of trying new foods over the 9 weeks, all children at individual tables in the lunchroom were visited on a weekly basis by the research assistants offering samples from brightly colored bowls. The research assistants offered enthusiasm and praise to empower and encourage students to try the samples. For example, research assistants exclaimed, “You are an adventurous eater!” or “Good for you for trying that!”. In addition, attractive presentation and alternating between fruits and vegetables increased interest. The program utilized several other strategies including exposure [38], sensory education [39,40,41], empowerment based on self-determination theory [42], and positive peer pressure based on social identity theory [43]. Sensory education was encouraged, inviting children to use all 5 senses to explore samples before choosing whether to eat them [44]. Empowerment included side-stepping power struggles [45] and allowing for participant choice [46]. Social identity included peer modeling [47], priming with positive descriptions/enthusiastic presentation [48], and praise [49]. Inviting the students to be “adventurous eaters” encouraged self-categorization with a social identity that inspires positive eating behaviors, a strategy shown to have benefits in food intake and other health behaviors [50].

All data were managed and analyzed using SAS 9.4 [51]. The FVNI responses were coded as shown in Appendix A, Table A1. A food neophobia scale (FNS) was computed using recoded responses to each of the 20 FVNI questions. The FNS ranged from a potential minimum of 0 (less neophobic) through a potential maximum of 62 (more neophobic). The 6 questions coded (1) “a lot” through (4) “not at all” and the 12 questions coded (1) “definitely” through (4) “definitely not” were recoded to values of 0 through 3. The neophobia coding for the 2 questions coded (0) “never” through (4) “at least 4 times” were reversed: (4) “at least 4 times” was coded as 0 and (0) “never” was coded as 4 so that the lower value represented less neophobic, and the higher value represented more neophobic. The scales were checked for normality for each school and each survey administration time (weeks 1 and 9) using probability plots and Shapiro-Wilk W. The Shapiro-Wilk W ranged from 0.90 to 0.96 indicating that the distributions of the FNS for each school and point in time did not significantly depart from normal. Heterogeneity of variance of the FNS comparing the intervention to the control school was assessed using Bartlett’s Test. The p-value for Bartlett’s was 0.51 at week 1 and 0.57 at week 9, indicating that the variances were homogeneous.

The FNS analysis explored within schools, between schools, and overall differences to answer the research questions listed below. Within school differences were compared using a paired *t*-test. Between school differences were compared using an independent samples *t*-test. Overall pre- and post-intervention differences controlling for school type were measured using repeated-measures analysis of variance (ANOVA).

- At week 1, baseline, did the FNS vary significantly when comparing the intervention school to the control school?
- At week 9, the end of the intervention period, did the FNS vary significantly when comparing the intervention school to the control school?
- Within the intervention school, did the FNS vary significantly when comparing survey results from week 1 to results from week 9?
- Within the control school, did the FNS vary significantly when comparing survey results from week 1 to results from week 9?
- Comparing the intervention school to the control school, did the FNS vary between week 1 and week 9, controlling for school type?

Observer counts were used to calculate percentages for each food item and each taste test. Data were compared between schools for each observer count percentage using chi-square. In addition, the absolute difference in the percentages of children who took, tasted, finished, and asked for seconds of the samples at the intervention school less the control school were used to quantify the “neophobia gaps” between the schools as well as the width of those gaps (“neophobia width gap”) between the 2 schools. 

## 3. Results

The analysis of the collected data had two distinct components: Food Neophobia Scale Analysis and Oral Survey and Behavioral Observation Analysis.

### 3.1. Food Neophobia Scale Analysis

The sample size for the FVNI was too small for individual items to have statistically significant results. The FVNI items were grouped and recoded as shown in Appendix A, Table A1 to create the FNS as described in the previous section. Table 2 shows the results of statistical analyses addressing each of the five research questions, the overall effect of time (pre- versus post-test), the interaction of time and school type (intervention versus control). The results of the independent samples *t*-tests do not reveal significant differences in the mean FNS scores comparing the intervention school to the control school at week 1 or at week 9. Paired *t*-tests reveal that there was a statistically significant decrease in the mean FNS score for the intervention school (decrease 3.0 scale points, *p* = 0.04), but not for the control school (decrease 2.0 scale points, *p* = 0.08). A repeated measures ANOVA revealed a statistically significant overall effect of time (*p* = 0.006), but not a significant interaction between school type and time (*p* = 0.57). These results suggest that time was the significant factor related to changes in FNS scores and that, although promising, the data do not show that the intervention was a significant factor in the FNS score change.

### 3.2. Oral Survey and Behavioral Observation Analysis

Both the intervention school and the control school classroom groups were offered the same food items (1 vegetable and 1 fruit) at the beginning and end of the 9-week study period. Table 3 shows the chi-square comparisons between the intervention and the control school for each food item and each oral survey response measuring attitude using raised hands (“had it before” and “liked it”) and observed behavior (“took it”, “finished it” and “took another”). At both pre- and post-intervention, there were no statistically significant differences in responses to the oral survey measuring attitude comparing the intervention school to the control school. Statistically significant differences were observed pre-intervention between the schools for behavior, specifically finishing the vegetable (arugula) and the fruit (pear) and for taking another sample of each food item (all with *p* < 0.001). For each of the pre-intervention items, the proportion for each behavior (“finished it”, “took another”) was higher among those in the control school compared to the intervention school. Post-intervention, these behaviors were not statistically significantly higher in the control school versus the intervention school for both the vegetable (spinach) and the fruit (green apple). Pre-intervention, the proportion who took both the vegetable and the fruit did not vary between the intervention and control schools. Post-intervention, the proportion taking the vegetable was statistically significantly higher among those in the intervention school compared to the control school (*p* = 0.007). Likewise, the proportion taking the fruit was higher in the intervention school compared to the control school (*p* < 0.001). The proportion tasting the vegetable was higher in the control school versus the intervention school (*p* = 0.009)

Appendix A, Table A2 shows the absolute differences for each oral survey question and observed behavior for each food item type (vegetable or fruit) within schools at pre- and post-intervention. The final two columns, shown in Table 4, quantify the neophobia gap (NG) and NG width observed between schools pre- and post-intervention. Additional statistical tests were not conducted for the information in Appendix A, Table A2 and Table 4 as the information in the table is intended only to show the magnitude, direction of absolute differences within each school and the neophobia gap width between schools. Positive absolute differences indicate the proportion was higher post-intervention compared to pre-intervention and vice versa. In the intervention school, the proportion of students raising hands for all the oral survey questions or displaying the observed behaviors was higher at the post-intervention food tasting for the vegetable and the fruit, except for tasting the vegetable and the fruit. In the control school, the proportion of students raising hands for all the oral survey questions or displaying the observed behaviors was higher at the post-intervention food tasting for the vegetable and the fruit, except for taking and tasting both the vegetable and the fruit.

As shown in Appendix A, Table A2, the data reveal small gap widths (<1 percentage point) in attitude for two vegetable oral survey questions (“had it before” and “liked it”) and one fruit behavior (“tasted it”). This indicates very small or no differences in the proportion noted for each attitude or behavior comparing the intervention school to the control school pre- and post-intervention. Moderately sized gap widths (1–10 percentage points) were observed for one vegetable behavior (“tasted it”) and two fruit attitudes items (“had it before” and “liked it”). Several of the neophobia gap widths are relatively large (>10 percentage points), indicating large shifts in the neophobia gap comparing the intervention school to the control school. The relatively large NG widths were observed for three vegetable and three fruit behaviors (“took it”, “finish it” and “took another”). For the vegetable, the NG width for “took it” is 20.8 percentage points. This large gap width stems from a −6.1 pre-intervention percentage point difference between the control school and the intervention school. At post-intervention, the NG for this item was 14.7 resulting in a net difference of 20.8 percentage points due, in large part, to the proportion in the control school declining by 18.7 percentage points, comparing pre- to post-intervention. For those observed finishing the vegetable, note that the proportion increased in the intervention school from 41% to 65.4%; whereas the proportion only increased by 8.3 percentage points in the control school. Similar patterns were observed for “took another” vegetable and three observed behaviors for the fruit (“took it”, “finish it” and “took another”).

In summary, analysis of the FNS, data revealed that there were no statistically significant differences in mean FNS comparing the control and intervention schools at baseline and at the conclusion of the intervention. Within the intervention school, the data revealed there was a statistically significant decrease in the mean FNS score over the course of the intervention, indicating a lower level of food neophobia. A statistically significant decrease was not observed in the control school. Using repeated measures ANOVA to examine the combined effect of school type (IS versus CS) and time, time was revealed to be the significant factor to the decrease in FNS, but the data do not suggest that the intervention was a significant factor regarding the decrease in the FNS score. Data from the oral survey responses and observed behaviors during the fruit and vegetable tastings show that, before the intervention, the IS and CS were similar for all but two behaviors: finishing the food item and taking another sample, which were significantly higher in the CS compared to the IS. When examining the neophobia gaps regarding the oral survey and observed behaviors, there were large neophobia gaps for three behaviors comparing the IS to the CS: taking the sample, finishing the sample, and taking another sample.

## 4. Conclusions

The purpose of the program is to empower children to make more deliberate choices of food and to help them learn to be more open specifically to fruits and vegetables. A highlight of the intervention used for this study (taste testing and educational and psychosocial strategies during weeks 2–8) is that it focused on both attitudes and behaviors. The analysis revealed a positive association between the intervention and both attitudes and behaviors, but behaviors most clearly. There was some evidence of a change in attitude at the intervention school. The FNS demonstrated that at the end of the intervention (week 9), there was a statistically significant difference within the intervention school between the pre- and post-tests, but not at the control. It is possible that over the course of a school year, exposed to other children’s food choices in a cafeteria, neophobic attitudes begin to wane across the board, but this study suggests that the waning process was accelerated by these food explorations because the significant difference between pre- and post-test occurred only at the intervention school.

In terms of observed behavior during the tastings and oral survey, the students at the control school began less food neophobic than at the intervention school. However, by the end of the intervention, that seems to have reversed: the higher percentage of students who took both items at post-test at the intervention school is statistically significant. Children at the intervention school were much more likely at the post-test to accept a sample of the food of the week, finish the sample, and take seconds than children at the control school by a large margin. Furthermore, the differences in the neophobia gaps widened both within each school and between the two schools; these are relevant findings. This program made a measurable difference in terms of behavior. Other studies similarly suggest that overall, there are moderate effects on fruit and vegetable consumption [22] (d= 0.45, 95% CI 0.33–0.59) or fruit consumption [23] (d = 0.24, 95% CI 0.05,0.43) from school-based programs supporting other research on utilizing strategies on social modeling [52].

A second conclusion examines the attitude and behavior data together. The attitude, as measured by the FNS, changed significantly only at the intervention school, and the oral survey data was mixed, but there was clear relevant change in behavior at the intervention school. This difference between reported attitude and observed behavior suggests that it is important to not rely solely on surveys because reported attitude and observed behavior do not always correlate, a finding shared with another similar study [53]. 

## 5. Discussion

There is not yet a clear consensus about whether including educational components in addition to increasing availability of fresh produce at school raises fruit and vegetable consumption. One study comparing a free distribution of fruits and vegetables to a school versus a multicomponent program involving education in the classroom and parent participation, the distribution program was found to be superior [54]. However, another study suggests that experiential learning activities made a difference to vegetable consumption [55]. A review of a wide variety of psychological and educational strategies [56] suggests that the choice of strategy employed makes a difference, and some appear to have more of an impact than others, which is why the strategies used in this intervention were tested in a pilot and deliberately employed. This study offers further evidence that an education component does impact attitude and behavior.

There is a suggestion in the data that in particular the elements of the program that invited children to take an exploratory approach to new foods and to feel empowered to choose whether or not to eat the sample may have had an impact. The data on how many students who took the sample and tasted it show a pattern. While for arugula, pears and apples, all students at both schools who took a sample, tasted it, students only at the intervention school accepted spinach but then did not taste it. On the surface, this may seem like more neophobic behavior at the intervention school to take a sample but refuse to eat it. However, in the context of the psychosocial and educational strategies of the intervention, it suggests an empowered yet exploratory attitude: children are willing to take something that they had not yet committed to eating. The intervention encouraged children to touch, smell, or lick a sample before consuming it, and these data may suggest that participants at the intervention school had internalized that lesson, and could also have led to more students choosing to finish the sample and ask for seconds at post-test than pre-test at the intervention school. This finding is similar to other studies on the effects of sensory education [39,40,44,57].

Although there were positive findings from this intervention, a few limitations existed. There are still open questions about whether the attitude and behavior changes extend outside the intervention context and whether it lasts beyond the intervention time. While this research did not examine these questions, the USDA’s Fresh Fruit and Vegetable Program (FFVP) offers a fruit or vegetable twice a week to low-income schools [58]. This program has led to the participants selecting more fruits and vegetables outside of the school setting within homes and grocery store shopping, thus impacting behavior outside the intervention context. The question of whether the changes last over time needs more attention. In a larger scale longitudinal cohort study of a different program, Fruits and Vegetables Make the Mark (FVMM), Norwegian children were offered a nutrition education program, and some of the intervention schools also received free fruits [59]. At the 7-year follow up, those schools that received the additional free fruit had a larger effect size than those that did not [60]. However, at the 14-year follow up, there were no concerted effects found between this free program and the intake of fruits and vegetables [61]. Longitudinal interventions are called for to evaluate the long-term effects of these programs into adulthood. 

An additional limitation of this study is that the schools differ significantly by race and ethnicity. The sample size for this study was insufficient to conduct meaningful statistical comparisons controlling for race/ethnicity. School principals chose whether to participate and there was not an opportunity to select schools with similar demographic characteristics. There is a difference between the race/ethnicity of the schools in that the intervention school had a majority of non-Hispanic white students, while the control school had a majority of Hispanic students. However, this simple comparison of racial demographics cannot be used as a proxy for cultural differences because data were not collected that would indicate what variety there is among countries of origin for Hispanic students or how assimilated either population might be. The analyses were not adjusted for race/ethnicity, and so it is not possible to know whether differences are due to the intervention or to the demographics of the study groups. The absolute difference in the percent of students on free and reduced lunch between the schools can, on the other hand, be used as a proxy for social-economic status [62] and is a relevant finding. The socioeconomic difference could have impacted the level of food neophobia between the schools, as those students with a higher SES eat more fruits and vegetables [63,64,65]. 

A third limitation is that the samples used in this study were donated by the school’s food service provider, which meant that the researchers did not have complete control of the choices offered. In a future study, it would be helpful to isolate the effect of neophobic attitudes by offering foods to which the children had not been previously exposed.

This intervention of a weekly fruit and vegetable tasting using particular psychosocial and educational strategies seems to have had an impact on both attitude and behavior. The effect was most clearly seen in behavior. This leads to the observation that it is very important to test behavior as well as attitude. Self-reported surveys, even simple oral ones, do not always correlate with behavior.

The research implications of this study include the finding that attitude and behavior were not correlated, and so it is important for future research projects to consider employing observation of behavior in addition to reliance on surveys of attitude. The implications for practice are clear: data suggest that this is a cost-efficient program that is easy to implement in schools across the country, and it had a measurable effect reducing food neophobia as seen in both attitude and behavior. Offering bite size samples to a school of 500 children over a 2.5-h lunch period takes just 2 volunteers 3-3.5 h (including prep and clean up time) once a week. Because the samples were bite-sized, the cost of the produce (when it isn’t donated) is minimal—about $400/year for 12 weeks of programming. Both the financial cost and the volunteer time can easily be provided by many school PTAs.

## Figures and Tables

**Table 1:** Gender and race/ethnic characteristics of the intervention (*n* = 33) and control (*n* = 42) schools, 2017–2018 school year.

Characteristic	Intervention School Percentage ^1^	Control School Percentage ^1^	Fisher’s Exact Two-Sided *p*-Value
Gender			0.82
Boys	45.5	48.8	
Girls	54.6	51.2	
Race/Ethnicity			0.003
Non-Hispanic White	69.7	33.3	
Non-Hispanic Black	12.1	7.1	
Hispanic	15.2	57.1	
Other	3.0	2.4	

^1^ Percentages may not equal 100% due to rounding.

**Table 2:** Food neophobia scale (FNS) research question analysis statistics comparing the intervention school (IS) to the control school (CS), comparing week 1 to week 9, and examining interaction between school type (IS versus CS) and time (week 1 versus week 9).

Research Question	Comparison	Mean (SD)	*p*-Value
At week 1, did the FNS vary significantly when comparing the intervention school to the control school?	IS	42.8 (11.3)	0.64 ^1^
	CS	44.0 (11.0)	
At week 9, did the FNS vary significantly when comparing the intervention school to the control school?	IS	39.8 (11.0)	0.38 ^1^
	CS	42.0 (10.0)	
Within the intervention school, did the FNS vary significantly comparing week 1 to week 9?	Week 1	42.8 (11.3)	0.04 ^2^
	Week 9	39.8 (11.0)	
Within the control school, did the FNS vary significantly comparing week 1 to week 9?	Week 1	44.0 (10.1)	0.08 ^2^
	Week 9	42.0 (10.0)	
Did the FNS vary between week 1 and week 9, controlling for school type (intervention versus control)?			
Within subjects (time)			0.006 ^3^
Combined effect of school type and time (school time)			0.57 ^3^

^1^ Independent samples *t*-test. ^2^ paired *t*-test. ^3^ repeated measures ANOVA (analysis of variance).

**Table 3:** Between-schools oral survey responses and behavioral observations comparisons.

Response or Behavior	Intervention School: % Raised Hand or Had Behavior	Control School: % Raised Hand or Had Behavior	Between-Schools Chi-Square *p*-Value
Pre-Intervention			
Arugula	*n* = 190	*n* = 196	
Had it before	21.1	18.7	0.67
Took it	87.4	93.5	0.14
Tasted it ^1^	100.0	100.0	1.00
Finish it ^2^	41.0	68.0	<0.001
Liked it ^2^	31.3	43.0	0.10
Took another ^2^	24.1	61.0	<0.001
Pears	*n* = 190	*n* = 196	
Had it before	69.5	73.5	0.53
Took it	90.5	90.7	0.98
Tasted it ^1^	100.0	100.0	1.00
Finish it ^2^	57.0	87.6	<0.001
Liked it ^2^	86.0	83.5	0.63
Took another ^2^	60.5	85.6	<0.001
Post-Intervention			
Spinach	*n* = 209	*n* = 181	
Had it before	77.6	74.4	0.62
Took it	89.5	74.8	0.007
Tasted it ^1^	91.8	100.0	0.009
Finish it ^2^	65.4	76.3	0.13
Liked it ^2^	50.0	61.3	0.15
Took another ^2^	56.4	70.0	0.08
Green apples	*n* = 209	*n* = 181	
Had it before	98.0	92.3	0.07
Took it	100.0	80.4	<0.001
Tasted it ^1^	100.0	100.0	1.00
Finish it ^2^	93.7	97.7	0.19
Liked it ^2^	96.8	96.5	0.90
Took another ^2^	90.5	96.5	0.11

^1^ Among those who took a sample of the food item. ^2^ Among those who tasted the food item.

**Table 4:** Comparison of Neophobia Gap (see full data table in Appendix A, Table A2) Between the Intervention School (*n* = 95) and the Control School (*n* = 107).

	Neophobia Gap (NG)		NG Width
Response or Behavior	Between Schools		|Pre-Post|
	Pre	Post	
Vegetable	Arugula	Spinach	
Had it before	2.4	3.2	0.8
Took it	−6.1	14.7	20.8
Tasted it ^1^	0	−8.2	8.2
Finish it ^2^	−27	−10.9	16.1
Liked it ^2^	−11.7	−11.3	0.4
Took another ^2^	−36.9	−13.6	23.3
Fruit	Pears	Apples	
Had it before	−4	5.7	9.7
Took it	−0.2	19.6	19.8
Tasted it ^1^	0	0	0
Finish it ^2^	−30.6	−4	26.6
Liked it ^2^	2.5	0.3	2.2
Took another ^2^	−25.1	−6	19.1

^1^ Among those that took the food item. ^2^ Among those that tasted it.

## Data Availability

The authors will send de-identified data upon request.

## Appendix A

**Table A1:** The table below displays the Fruit and Vegetable Neophobia Instrument (FVNI) questions, grouped by coded response option categories. The questions and response options were used to develop the Food Neophobia Scale (FNS) for this study.

Questions with Coded Response Options: (1) a lot, (2) a little, (3) not very much, (4) not at all
1. How much do you like fruit?
2. When you try a new fruit for the first time, how much do you usually like it?
3. How much do you like tasting new fruits?
11. How much do you like vegetables?
12. When you try a new vegetable for the first time, how much do you usually like it?
13. How much do you like tasting new vegetables?
Questions with Response Options: (1) definitely, (2) probably, (3) probably not, (4) definitely not
4. Will you taste a fruit if you don’t know what it is?
5. Will you taste a fruit if it looks strange?
6. Will you taste a fruit if you have never tasted it before?
7. When you are at a friend’s house, will you try a new fruit?
8. When you are at school, will you try a new fruit?
9. When you are at home, will you try a new fruit?
14. Will you taste a vegetable if you don’t know what it is?
15. Will you taste a vegetable if it looks strange?
16. Will you taste a vegetable if you have never tasted it before?
17. When you are at a friend’s house, will you try a new vegetable?
18. When you are at school, will you try a new vegetable?
19. When you are at home, will you try a new vegetable?
Questions with Response Options: (0) never, (1) 1 time, (2) 2 times, (3) 3 times, (4) at least 4 times
10. How many times have you tried a new fruit since school started this year?
20. How many times have you tried a new vegetable since school started this year?

**Table A2:** Comparison of oral survey and behavioral observations pre/post percentages in each school and absolute differences between the intervention school (*n* = 95) and the control school (*n* = 107).

	Intervention School (IS)			Control School (CS)			Neophobia Gap (NG)		NG Width
Response or Behavior	Percentage ^1^		Within IS ^2^	Percentage ^1^		Within CS ^2^	Between Schools (IS-CS)		|Pre-Post|
	Pre	Post	Absolute Difference	Pre	Post	Absolute Difference	Pre	Post	
Vegetable	Arugula	Spinach		Arugula	Spinach		Arugula	Spinach	
Had it before	21.1	77.6	56.5	18.7	74.4	55.7	2.4	3.2	0.8
Took it	87.4	89.5	2.1	93.5	74.8	−18.7	−6.1	14.7	20.8
Tasted it ^3^	100	91.8	−8.2	100	100	0	0	−8.2	8.2
Finish it ^4^	41	65.4	24.4	68	76.3	8.3	−27	−10.9	16.1
Liked it ^4^	31.3	50	18.7	43	61.3	18.3	−11.7	−11.3	0.4
Took another ^4^	24.1	56.4	32.3	61	70	9	−36.9	−13.6	23.3
Fruit	Pears	Apples		Pears	Apples		Pears	Apples	
Had it before	69.5	98	28.5	73.5	92.3	18.8	−4	5.7	9.7
Took it	90.5	100	9.5	90.7	80.4	−10.3	−0.2	19.6	19.8
Tasted it ^3^	100	100	0	100	100	0	0	0	0
Finish it ^4^	57	93.7	36.7	87.6	97.7	10.1	−30.6	−4	26.6
Liked it ^4^	86	96.8	10.8	83.5	96.5	13	2.5	0.3	2.2
Took another ^4^	60.5	90.5	30	85.6	96.5	10.9	−25.1	−6	19.1

^1^ Raised hand or had observed behavior. ^2^ post-intervention minus pre-intervention. ^3^ among those that took the food item. ^4^ among those that tasted it.
